# Platelet-Rich Plasma in Interstitial Cystitis/Bladder Pain Syndrome: A Systematic Review and Meta-Analysis

**DOI:** 10.34172/apb.025.45444

**Published:** 2025-09-03

**Authors:** Sakineh Hajebrahimi, Fateme Tahmasbi, Elham Jahantabi, Gholamreza Hosseinpour, Rajesh Taneja, Hanieh Salehi-Pourmehr

**Affiliations:** ^1^Research Center for Evidence-Based Medicine, Iranian EBM Centre: A JBI Centre of Excellence, Faculty of Medicine, Tabriz University of Medical Sciences, Tabriz, Iran; ^2^Department of Urology, Tabriz University of Medical Sciences, Tabriz, Iran; ^3^Social Determinants of Health Research Center, Health Management and Safety Promotion Research Institute, Tabriz University of Medical Sciences, Tabriz, Iran; ^4^Student Research Committee, Tabriz University of Medical Sciences, Tabriz, Iran; ^5^Urology and Robotic Surgery Indraprastha Apollo Hospitals, New Delhi, India; ^6^Medical Philosophy and History Research Center, Tabriz University of Medical Sciences, Tabriz, Iran

**Keywords:** Platelet-rich plasma, Interstitial cystitis/bladder pain syndrome, PRP, IC/BPS, Systematic review

## Abstract

**Purpose:**

This systematic review aims to critically evaluate the safety and efficacy of PRP therapy in managing interstitial cystitis/bladder pain syndrome (IC/BPS).

**Methods:**

Two researchers independently searched related Databases and collected all studies from inception to December 5, 2023. Outcome indicators of symptom relief were pain scores self-assessment using the VAS system, IC symptoms using the O’Leary-Sant score (OSS), urinary frequency, nocturia, post-void residual (PVR), voided volume, and functional bladder capacity.

**Results:**

Among 372 retrieved articles, 13 studies, including 426 patients, were included. The pain of patients decreased significantly after treatment with platelet-rich plasma (PRP) compared to the baseline values (MD: -1.93, 95% CI: -2.28, -1.58). All subgroup analyses revealed a decrease in VAS scores after PRP injection. IC symptoms using OSS, and ICSI decreased significantly after treatment.

**Conclusion:**

PRP therapy as a new and successful course of treatment may be a novel therapeutic approach in IC/BPS cases. More study with the control arm is required to enhance treatment regimens for this difficult condition and to better understand the mechanisms of action of PRP in IC/BPS.

## Introduction

 Interstitial cystitis/bladder pain syndrome (IC/BPS) imposes a profound clinical and socioeconomic burden on patients, with symptoms such as chronic pelvic pain, urinary urgency/frequency, and nocturia significantly impairing quality of life (QoL).^1^ Epidemiological studies report a prevalence of 1–5% in women and 0.1–0.3% in men, though underdiagnosis is common due to symptom overlap with other urological conditions.^2^ Patients often experience debilitating pain comparable to rheumatoid arthritis or endometriosis, leading to reduced work productivity, social isolation, and high rates of comorbid depression and anxiety. Despite its severity, IC/BPS remains a therapeutic challenge. First-line treatments (e.g., oral amitriptyline, pentosan polysulfate, and intravesical instillations) exhibit variable efficacy, with 40–60% of patients failing to achieve sustained symptom relief.^3^ Invasive options (e.g., hydrodistension, neuromodulation) are reserved for refractory cases but carry risks of complications and inconsistent long-term benefits.^4-7^ The pathophysiology of IC/BPS is multifactorial, involving urothelial dysfunction, neurogenic inflammation, and fibrosis, yet no therapy directly targets these mechanisms.^6,8^ This unmet need underscores the urgency for novel treatments like platelet-rich plasma (PRP), which may modulate inflammation, promote tissue repair, and restore bladder barrier integrity through growth factors such as VEGF and PDGF. The therapeutic rationale for PRP in IC/BPS derives from its unique capacity to target the condition’s multifactorial pathophysiology through regenerative and immunomodulatory mechanisms. As an autologous concentration of platelets, growth factors, and cytokines, PRP may promote urothelial restoration by stimulating proliferation of damaged umbrella cells through epidermal growth factor (EGF) and transforming growth factor-beta (TGF-β) signaling, while its fibrin matrix provides structural support during tissue repair. Simultaneously, PRP appears to modulate neurogenic inflammation by vascular endothelial growth factor (VEGF)-mediated normalization of aberrant bladder angiogenesis and interleukin-1 receptor antagonist (IL-1RA)-mediated suppression of mast cell activation, potentially reducing neuronal hypersensitivity characteristic of IC/BPS.^9-11^ Furthermore, PRP’s balanced regulation of matrix metalloproteinases (MMPs) and tissue inhibitors of metalloproteinases (TIMPs) may reverse fibrotic remodeling by restoring extracellular matrix homeostasis, while hepatocyte growth factor (HGF) counteracts TGF-β1-driven myofibroblast activation in the detrusor muscle.^12-14^ This multifaceted action profile positions PRP as a potentially disease-modifying therapy capable of concurrently addressing IC/BPS’s core pathological triad of epithelial dysfunction, neuroinflammatory dysregulation, and progressive fibrosis, a distinct advantage over current single-target approaches that often provide only symptomatic relief.^15,16^ IC/BPS significantly impacts patients’ quality of life, with many experiencing chronic pain and urinary dysfunction that remains refractory to conventional treatments such as oral medications (e.g., amitriptyline, pentosan polysulfate) and intravesical therapies. The lack of consistently effective treatments underscores the need for novel therapeutic approaches like PRP, which may address underlying pathological mechanisms such as chronic inflammation, urothelial dysfunction, and fibrosis. PRP has emerged as a promising regenerative therapy due to its ability to release growth factors (e.g., PDGF, VEGF) and cytokines that modulate inflammation and promote tissue repair. This systematic review and meta-analysis aim to offer objective insights into the therapeutic potential of PRP in managing IC/BPS

## Methods

###  Literature search and article selection

 PubMed, Web of Science, Embase, Medline, CINAHL, Cochrane Library, Scopus, Google Scholar, and ProQuest Databases were searched electronically, and all studies from the date database created up until December 5, 2023, gathered using “Interstitial cystitis/bladder pain syndrome”, “Prostatitis”, “Pelvic pain”, and “platelet-rich plasma” keywords. We conducted a manual search for additional relevant literature, including studies referenced in systematic reviews and meta-analyses, to supplement our search efforts. The search strategy in PubMed is presented in Supplementary file, Table S1.

###  Inclusion and exclusion criteria

 The literature screening process for this study had predetermined inclusion and exclusion criteria to ensure a rigorous and systematic approach. Patients diagnosed with IC/BPS through clinical examination who underwent PRP injection treatment and had outcome measures such as pain scores assessed using the VAS system, IC symptoms, functional activity assessed through scales like Urodynamic assessment, bladder diary, and post-voiding residue measurement were included in the study. The study designs considered were trials or observational studies, such as cohorts or case controls. Exclusion criteria comprised animal studies, injection of other medications within the past year, unavailability of access to full-text, and missing data.

###  Study selection

 For study selection, all identified studies were imported into EndNote version/20 for document management, and duplicates were eliminated. Then, title and abstract screening were conducted by two independent authors. Each potentially relevant study was then reviewed in full text and assessed for inclusion by the authors independently. Any discrepancies were resolved through consensus under the guidance of the principal investigator.

###  Data extraction

 Two reviewers autonomously gathered the treatment details from the literature incorporated in the study. Specific treatment parameters like injection site, dosage, and frequency were meticulously documented for the intervention technique. In instances where the reviewers encountered ambiguous or intricate data extractions from the literature, they reached out to the original authors to acquire comprehensive experimental information.

###  Quality assessment 

 Two researchers independently evaluated the Cochrane Risk of Bias tool for potential bias in eligible studies, resolving any differences through discussion and consensus or by the third reviewer.

###  Quality of Evidence Assessment (GRADE)

 To evaluate the certainty of the evidence and potential for bias in our meta-analysis, we employed two key methodologies. Firstly, we used the Grading of Recommendations Assessment, Development and Evaluation (GRADE) framework to systematically assess the certainty of evidence for each outcome. This involved starting with a high certainty of evidence, characteristic of randomized controlled trials, and then downgrading the rating based on five factors: risk of bias, inconsistency, indirectness, imprecision, and publication bias. The final certainty of evidence was categorized as high, moderate, low, or very low. Our rationale for each rating is documented in the Summary of Findings (SoF) table.

###  Publication bias 

 Secondly, we assessed for the presence of publication bias by visually inspecting funnel plots for outcomes with at least 10 included studies. Funnel plots, which graphically represent the standard error of the mean difference against the mean difference, were analyzed for symmetry. Asymmetry in the plot can suggest that smaller studies with non-significant or negative findings may be underrepresented or unpublished.

###  Statistical analysis

 Statistical analysis using Review Manager 5.4.1 was employed to assess heterogeneity among the studies. Heterogeneity was quantified using I^2^.^17^ For this meta-analysis, a fixed effects model was utilized when I^2^ < 50% and a random effects model for I^2^ > 50%.^18^

## Results

###  Results of the literature search

**Figure 1:**
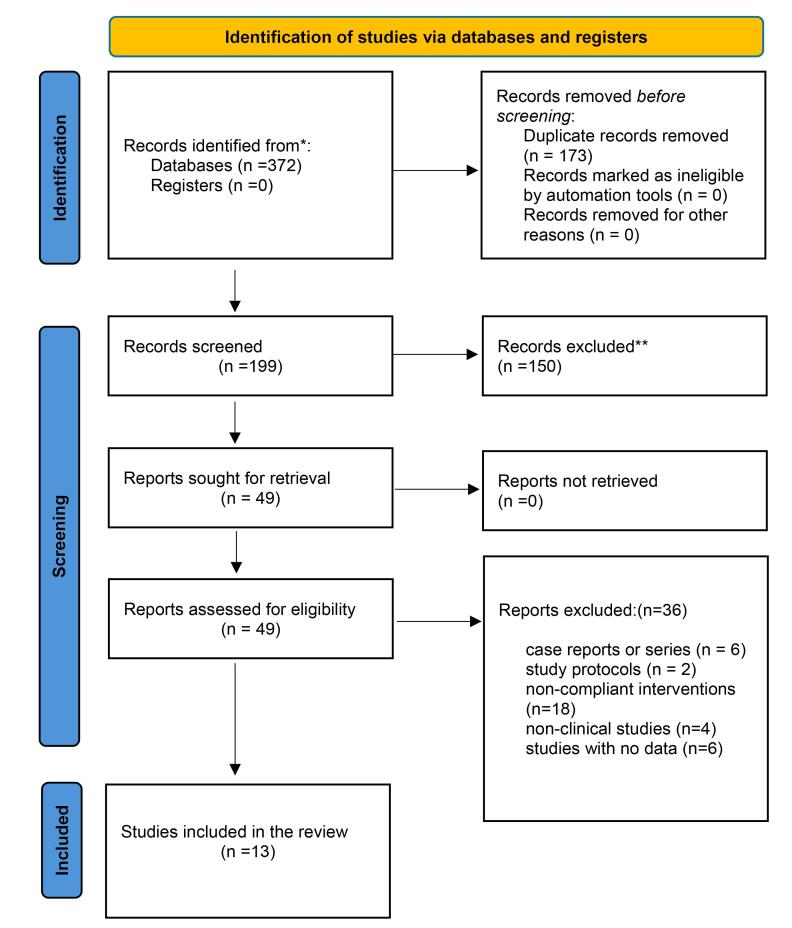
PRISMA flow diagram

 Through an electronic search, initially, 372 studies were identified. One hundred ninety-nine studies remained after duplicate removal. In the titles and abstracts screening, two reviewers eliminated 150 studies that had no bearing on the subject. After carefully going over the remaining 49 papers, 36 were eliminated for a variety of reasons, including not being clinical trials, case reports, or case series, research protocols, non-compliant interventions, or studies lacking data. Ultimately, the final review comprised thirteen acceptable investigations^19-31^ (including 426 patients) for qualitative evaluation, and eight studies for quantitative analysis (Figure 1).

###  Characteristics of included studies

 Supplementary file, Table S2 show the study results included in our systematic review, Nanofat grafts + PRP were injected once, and in another study, one injection was performed only.^24^ The patient’s whole blood was applied for PRP preparation. In addition, females were the most candidates for PRP injection. The included studies had no control arms except for the study of Jhang 2023,^23^ the patients in the control arm received BoNT-A injection. Studies reported that the range of follow-up was one to six months.

###  Results of the quality assessment

**Figure 2:**
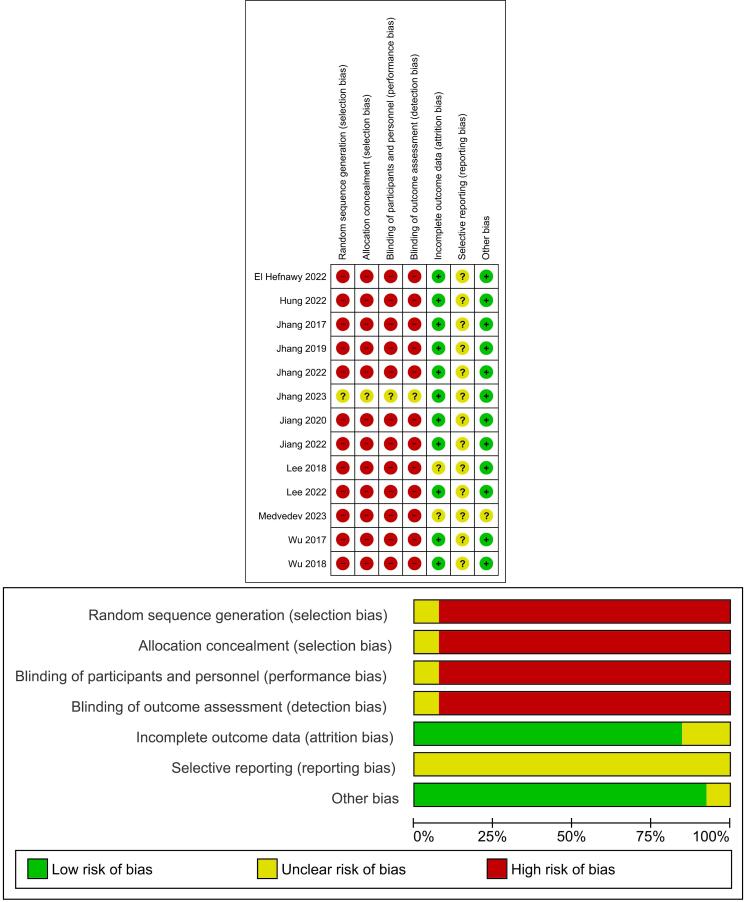
(a) Risk of bias graph: review authors’ judgements about each risk of bias item presented as percentages across all included studies; (b) Risk of bias summary: review authors’ judgments about each risk of bias item for each included study

 As most of the studies lacked a control arm, all risk-bias assessments were rated as unclear or high risk. Mostly, the clinical trials that were included had a moderate to high risk of bias. The risk of bias of the included studies was assessed using the Cochrane Handbook for Systematic Reviews of Interventions. The overall quality of the studies was found to be mixed, with many studies presenting a high or unclear risk of bias in several key domains. The findings are summarized below and in the risk of bias graph (Figure 2).

*Random sequence generation (selection bias):* The majority of studies were assessed as having a low risk of bias in this domain, indicating that participants were randomly assigned to groups. However, a notable number of studies were at high risk or unclear risk of bias, which could affect the comparability of the groups at baseline.

*Allocation concealment (selection bias):* This domain was a major source of concern. Most studies were rated as having an unclear risk of bias, suggesting that the method used to conceal the allocation sequence was either not reported or was insufficient to prevent foreknowledge of the group assignment.

*Blinding of participants and personnel (performance bias):* This was the most prevalent source of high risk of bias. A very high percentage of studies were rated as being at high risk of performance bias, indicating that participants and the personnel delivering the intervention were aware of the treatment assignment. This is expected given the nature of the PRP injection procedure.

*Blinding of outcome assessment (detection bias):* This domain also showed a significant risk of bias. Many studies were rated as having a high risk of detection bias, suggesting that the outcome assessors were not blinded to the treatment groups, which could have led to a biased assessment of the results.

*Incomplete outcome data (attrition bias):* The majority of studies were rated as having a low risk of bias for incomplete outcome data, which suggests that study dropouts and exclusions were handled appropriately and did not significantly impact the results.

*Selective reporting (reporting bias):* Almost all studies were rated as having an unclear risk of bias in this domain. This indicates that it was difficult to determine from the study protocols or publications whether all expected outcomes were reported.

*Other bias:* The majority of studies were found to have a low risk of bias in this category.

###  Results of statistical analysis

####  Urinary and urodynamic outcomes

 The meta-analysis of included studies assessed the effect of PRP on several urinary and urodynamic parameters.

**Figure 3:**
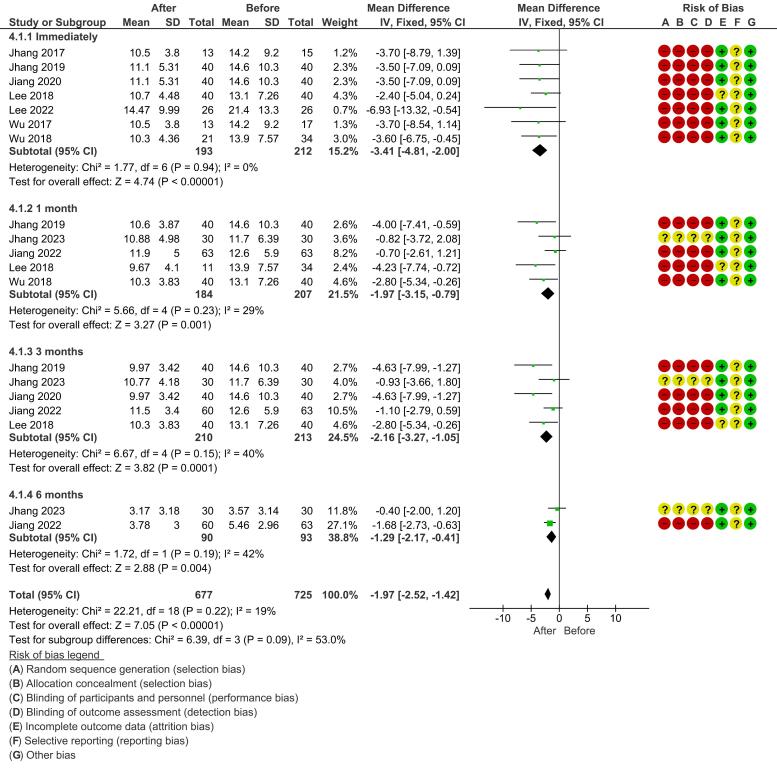
The effect of PRP on Urinary frequency at different time points

*Frequency:* A fixed-effect model was used for the immediate and 1-month follow-up periods due to low heterogeneity (I^2^ = 0%) and non-significant χ2 values. The overall effect for immediate follow-up showed a statistically significant reduction in urinary frequency (mean difference, -3.41 [-4.81, -2.00]; *P* < 0.00001). This significant reduction was also observed at the 1-month follow-up (mean difference, -1.97 [-3.15, -0.79]; *P* = 0.001). At 3 months, the fixed-effect model showed a significant reduction (mean difference, -2.43 [-3.94, -0.92]; *P* = 0.001) (Figure 3).

**Figure 4:**
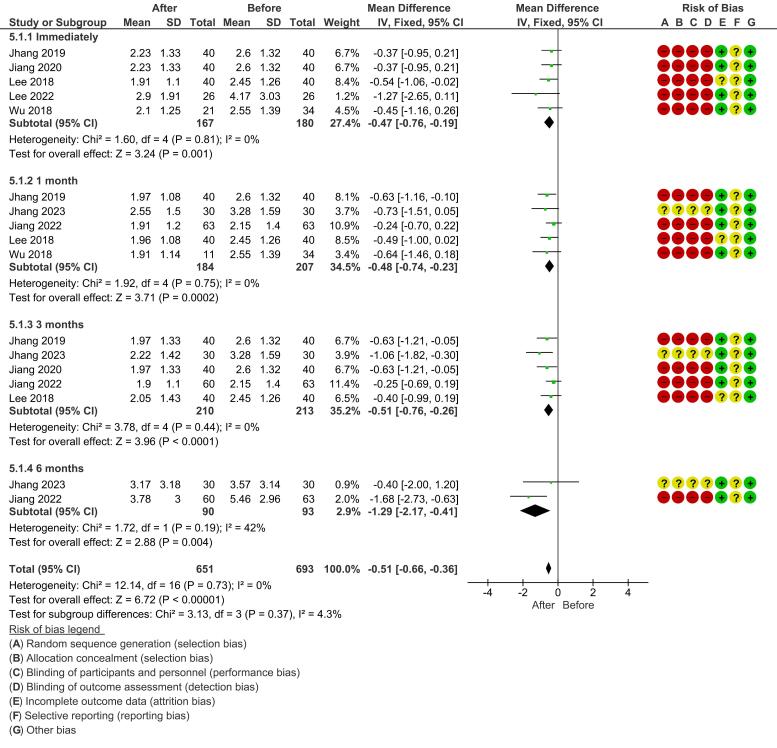
The effect of PRP on Nocturia at different time points

*Nocturia:* A fixed-effect model was also used for nocturia. A significant reduction was found immediately after treatment (mean difference, −0.47 [-0.76, -0.19]; *P* = 0.001). The significant effect was maintained at 1 month (mean difference, −0.48 [-0.74, -0.23]; *P* = 0.0002) and at 3 months (mean difference, −0.51 [-0.76, -0.26]; *P* < 0.0001). All sub-analyses for nocturia showed very low heterogeneity (I^2^ = 0%) (Figure 4).

**Figure 5:**
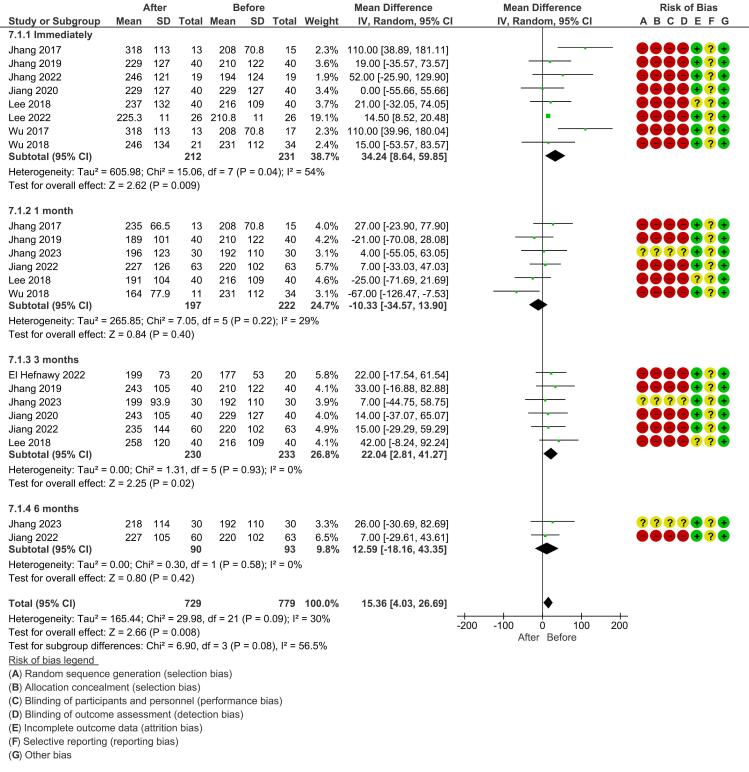
The effect of PRP on Voided volume at different time points

*Voided volume:* For voided volume, a random-effects model was used due to significant heterogeneity across the studies, as indicated by an I^2^ value of 54% for the immediate follow-up (*P* = 0.04). The pooled analysis at the immediate follow-up showed a significant increase in voided volume (mean difference, 34.24 [8.64, 59.85]; *P* = 0.009). However, at 1 month and 3 months, the results were not statistically significant, and heterogeneity was still present (I^2^ = 29% and I^2^ = 0%, respectively) (Figure 5).

**Figure 6:**
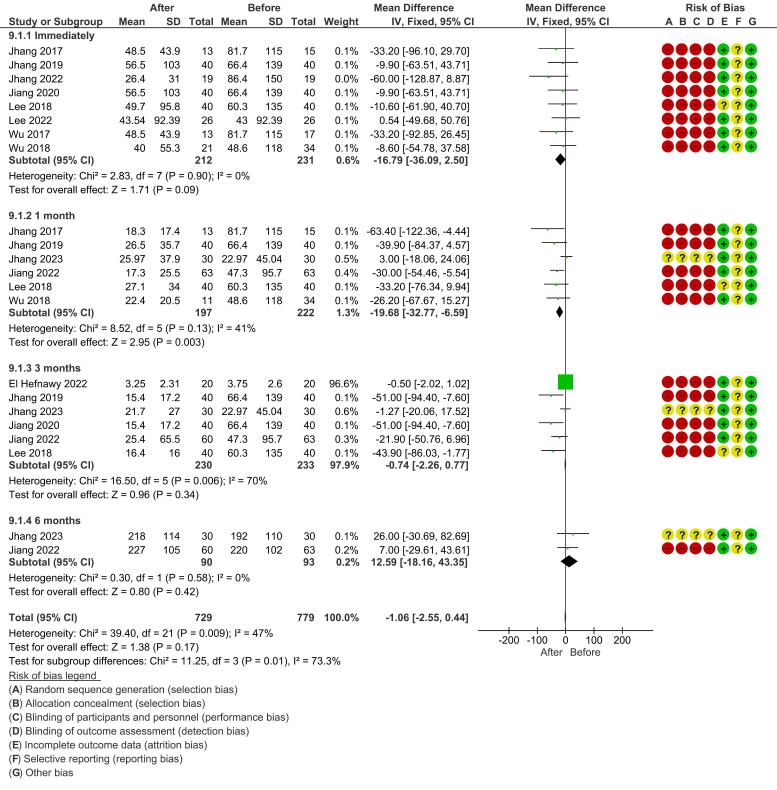
The effect of PRP on Post void residual at different time points

*Post-void residual (PVR):* Due to high heterogeneity across studies, a random-effects model was used for all time points. The meta-analysis for immediate post-treatment PVR showed a non-significant mean difference (mean difference, −16.79 [−36.09, 2.50]; *P* = 0.09) with no heterogeneity (I^2^ = 0%). At 1 month, the pooled effect was a significant reduction (mean difference, −19.68 [−32.77, −6.59]; *P* = 0.003), though heterogeneity was moderate (I^2^ = 41%). At 3 months, the reduction was not statistically significant (mean difference, −0.74 [−2.26, 0.77]; P = 0.34). The 6-month follow-up showed a non-significant but positive mean difference (Figure 6).

*Maximum flow rate (Qmax):* A random-effects model was used for all time points due to significant heterogeneity. The immediate post-treatment analysis showed a non-significant increase in Qmax (mean difference, 3.29 [−0.48, 7.05]; *P* = 0.09), with moderate heterogeneity (I^2^ = 68%). A similar non-significant trend was observed at 1 month. However, in 3 months, there was a significant increase in Qmax (mean difference, 7.39 [4.24, 10.54]; *P* < 0.00001) with low heterogeneity (I^2^ = 0%) (Supplementary file, Figure S1).

*Functional bladder capacity (FBC):* A fixed-effect model was used due to low heterogeneity for all time points. At the immediate and 1-month follow-ups, the meta-analysis showed a significant increase in FBC (mean difference, 41.04 [19.47, 62.62]; *P* = 0.0002 for both). The effect was also significant for 3 months, with a mean difference of 45.10 [23.51, 66.68]; *P* < 0.0001 (Supplementary file, Figure S2).

*Cystometric Bladder Capacity (CBC):* A random-effects model was used for the immediate and 1-month follow-up due to high heterogeneity (I^2^ = 82% and I^2^ = 72%, respectively). The immediate follow-up showed a non-significant increase in CBC (mean difference, 43.96 [-10.35, 98.27]; *P* = 0.11). At 1 month, the effect was also not significant (mean difference, −71.98 [-103.31, −40.62]; *P* < 0.00001). At 3 months, a fixed-effect model was used due to low heterogeneity (I^2^ = 0%) and the result was also not significant (mean difference, −14.03 [−44.51, 16.46]; *P* = 0.37) (Supplementary file, Figure S3).

###  Symptom outcomes

 The analysis of patient-reported symptoms also used fixed-effect and random-effect models as appropriate.

*Visual analogue scale (VAS):* A fixed-effect model was used for the immediate follow-up due to low heterogeneity (I^2^ = 0%). The analysis showed a significant reduction in VAS scores (mean difference, −1.93 [−2.36, −1.50]; *P* < 0.00001). A random-effects model was used for the 1-month and 3-month follow-ups due to significant heterogeneity (I^2^ = 57% and I^2^ = 73%, respectively). Both of these follow-ups also showed significant reductions in VAS scores (Supplementary file, Figure S4).

*Interstitial cystitis symptom index (ICSI):* A random-effects model was applied due to high heterogeneity. Immediately post-treatment, the meta-analysis demonstrated a significant reduction in ICSI scores (mean difference, −2.99 [-3.70, -2.27]; *P* < 0.00001). The heterogeneity was moderate (I^2^ = 35.4%). At 1 month, the significant reduction was maintained (mean difference, −2.82 [-3.55, -2.09]; *P <*0.00001) with low heterogeneity (I^2^ = 0%). At 3 months, a significant reduction was also observed (mean difference, −4.45 [-5.45, -3.45]; *P* < 0.00001) with low heterogeneity (I^2^ = 0%) (Supplementary file, Figure S5).

*Interstitial cystitis pain index (ICPI):* A random-effects model was used due to moderate heterogeneity (I^2^ = 35.1%, *P* = 0.17). The immediate post-treatment analysis showed a significant reduction in ICPI scores (mean difference, −2.82 [-3.55, -2.09]; *P* < 0.00001). At 1 month, this effect remained significant (mean difference, −3.10 [-3.84, -2.35]; *P* < 0.00001) with low heterogeneity (I^2^ = 0%). The 3-month follow-up also showed a significant reduction (mean difference, −4.20 [-5.08, -3.31]; *P* < 0.00001) with low heterogeneity (I^2^ = 0%) (Supplementary file, Figure S6).

*O’Leary-Sant symptom score (OSS):* A fixed-effect model was used for the immediate follow-up due to low heterogeneity (I^2^ = 21%). The analysis showed a significant reduction in OSS (mean difference, −6.35 [−7.84, −4.87]; *P* < 0.00001). At 1 month, a random-effects model was used due to significant heterogeneity (I^2^ = 71%), and the reduction remained significant (mean difference, −7.19 [−8.96, -5.42]; *P* < 0.00001). The 3-month follow-up also showed a significant reduction in OSS (mean difference, −10.15 [−12.44, −7.86]; *P* < 0.00001), with high heterogeneity (I^2^ = 80%) (Supplementary file, Figure S7).

###  Patient-reported outcomes (PROs)

 Data on patient-reported outcomes beyond standardized scores were very limited. Specifically, a quantitative meta-analysis of the Global Response Assessment (GRA) was not possible due to a lack of consistent data reporting. Most studies included in the immediate follow-up analysis for GRA did not provide the necessary data for estimation, resulting in “Not estimable” results. The single study that did provide data showed a non-significant mean difference. Thus, a conclusive statement cannot be made based on the provided forest plots for the immediate outcome (Supplementary file, Figure S8).

###  Adverse event reporting results

 No related adverse reactions after PRP injections were reported in most studies, except discomfort with blood sample withdrawal in one study.^19^

###  Certainty of the evidence

 The overall quality of the included studies was found to be mixed, with a high or unclear risk of bias in several key domains, which significantly impacted our GRADE ratings.

 Our GRADE assessment found that the certainty of evidence for most outcomes was either low or very low. This was primarily driven by the high risk of bias in the included studies, particularly due to the lack of blinding of participants, personnel, and outcome assessors. For example, the certainty of evidence for urinary frequency was rated as low, primarily because the risk of bias from unblinded study personnel could have influenced the self-reported data. Similarly, the evidence for Qmax was also rated as low due to this same risk of bias and high heterogeneity at other time points.

 For other outcomes, the certainty of evidence was downgraded to very low due to a combination of high risk of bias and serious imprecision. The outcomes for PVR and CBC are prime examples; while some significant results were found, the wide confidence intervals in the pooled analysis made the true effect of the intervention highly uncertain. This lack of precision, combined with the prevalent risk of bias, led to the very low-GRADE rating. See Supplementary file, Table S3.

###  Results of publication bias assessment 

 In our evaluation for publication bias, a visual inspection of the funnel plots revealed a mixed pattern. The funnel plots for urinary frequency and nocturia appeared relatively symmetrical, suggesting a low risk of publication bias for these outcomes. Conversely, the plots for voided volume, PVR, Qmax, OSS, and FBC showed varying degrees of asymmetry. This observed asymmetry suggests a potential publication bias where studies with certain results (e.g., non-significant or negative findings for voided volume) may be underreported. Therefore, the results for these specific outcomes should be interpreted with caution.

## Discussion

 Our systematic review and meta-analysis of 13 studies and 426 patients suggest that PRP therapy may be a promising therapeutic approach for IC/BPS. We observed statistically significant reductions in patient-reported pain scores (VAS), IC symptom scores (OSS, ICSI, ICPI), and improvements in urinary parameters like frequency, nocturia, and functional bladder capacity (FBC). These findings indicate that PRP therapy may provide symptomatic relief for a challenging condition with limited effective treatments. Autologous PRP is emerging as a potential treatment option for IC/BPS due to its ability to promote tissue regeneration and reduce inflammation.^32^ Autologous PRP injections administered intravesically on multiple occasions were reported to reduce the symptoms of IC in earlier trials.^22,31^ Improvement in the subjective sense is likely to affect the clinical efficacy of an innovative treatment for IC/BPS. As a result, stronger data is required to demonstrate its therapeutic effectiveness. Our findings found that PRP injection therapy was effective in lowering VAS pain ratings, OSS, ICSI, ICPI, and PVR. In addition, it increased the FBC, voided volume, and Qmax. Although CBC was decreased after treatment, this change was not statistically significant.

 BoNT-A may inhibit inflammatory mediator release, while PRP can modulate inflammation in IC/BPS bladders. Repetitive injections of these treatments can reduce inflammation, promote tissue regeneration, and improve bladder health, leading to pain relief. BoNT-A has strong evidence for efficacy but may reduce detrusor contractility, unlike PRP. PRP may require more frequent injections for optimal results, but it offers potential therapeutic benefits for IC/BPS patients.^23,33^

 Our results indicate that PRP could be a valuable treatment option for pain management and symptom improvement in individuals with IC/BPS. This makes PRP a ‘potentially effective’ treatment in reducing the overall severity of IC/BPS symptoms. PRP treatment also resulted in improvements in various urinary parameters, suggesting that PRP may be effective in improving bladder function in individuals with IC/BPS.

 While the evidence supporting the use of PRP in IC/BPS is still limited and further research is needed to establish its efficacy and safety, early studies have shown promising results. Although frequency, nocturia, and FBC showed a significant improvement statistically, there is no general consensus among experts on whether this change is clinically meaningful or not. There is no precise information about patient-reported outcomes, and only the change related to GRA was reported, which was not analyzable. Therefore, there is no information available on whether these changes were meaningful for patients. Further studies should be conducted with a large number of patients and a control group to compare the therapeutic effects of PRP with other therapeutic methods, better understand the mechanisms of action of PRP in IC/BPS, and to optimize treatment protocols for this challenging condition.

###  Clinical meaningfulness of findings 

 While our meta-analysis showed a statistically significant reduction in VAS scores, it is crucial to consider the clinical meaningfulness of this change. A reduction of this magnitude may not be substantial enough for all patients to experience a meaningful improvement in their daily lives. Similarly, the statistical significance of other scores, such as OSS, needs to be interpreted in the context of individual patient experiences. This underscores the need for future studies to not only report statistical significance but also to define and measure clinically meaningful changes from a patient-centered perspective.

###  Study limitations and biases

 A major limitation of the included studies is the near-universal absence of a control group. With only one study using a comparator (BoNT-A), it is impossible to definitively distinguish the effects of PRP from placebo effects or the natural course of the disease. Given the subjective nature of IC/BPS symptoms, the placebo effect can be substantial. The high risk of bias identified in most studies further weakens our findings, as a lack of blinding and randomization could have influenced the reported outcomes.

###  Heterogeneity of PRP protocols

 The included studies exhibited a wide range of heterogeneity in PRP preparation, dosage (8-50 mL), frequency (one to multiple injections), and administration sites (e.g., submucosal, bladder wall). This lack of standardization makes it challenging to pool data and limits the generalizability of our findings. The optimal PRP protocol for IC/BPS treatment remains unclear, and future research must focus on defining a standardized approach to ensure replicable results.

###  Practical considerations

 Beyond efficacy and safety, the practical implementation of PRP therapy in a clinical setting must be considered. Issues such as the cost of the procedure, accessibility for patients, and the acceptability of the injection method are important factors that were not consistently reported in the literature. While autologous PRP is generally safe, these practical considerations will influence its adoption as a mainstream treatment.

###  Future directions

 To overcome the limitations of the current literature, future research on PRP for IC/BPS should prioritize well-designed, randomized controlled trials (RCTs). These studies should include a placebo control group (e.g., saline injection) to isolate the true therapeutic effect of PRP. Standardized protocols for PRP preparation and administration are essential. Finally, future studies should focus on reporting robust patient-reported outcomes, including GRA and quality of life measures, to better capture the patient-centered nature of IC/BPS.

## Conclusion

 The results of this systematic review and meta-analysis suggest that PRP therapy is a promising, albeit preliminary, therapeutic approach for managing the symptoms of IC/BPS. The therapy appears to be associated with statistically significant improvements in pain, urinary symptoms, and functional bladder capacity. However, these findings must be interpreted with caution due to significant limitations in the available literature. The high risk of bias, lack of control groups in most studies, and substantial heterogeneity in PRP protocols limit the clinical certainty of our conclusions. Further high-quality, randomized controlled trials with standardized treatment protocols and a focus on clinically meaningful patient-reported outcomes are urgently needed to validate PRP’s efficacy and safety for IC/BPS.

## Competing Interests

 None.

## Data Availability Statement

 Not applicable

## Ethical Approval

 The Research Ethics Committee of the Tabriz University of Medical Sciences approved the study (Grant No.73068; IR.TBZMED.REC.1403.058).

## Supplementary Files

Supplementary file contains Tables S1-S3 and Figure S1-S8.
