# Supplementary material for: Platelet-Rich Plasma in Interstitial Cystitis/Bladder Pain Syndrome: A Systematic Review and Meta-Analysis
Source: Adv Pharm Bull. 2025 Sep 3;15(3):521–32. doi: 10.34172/apb.025.45444 (PMC12703394; doi:10.34172/apb.025.45444)
Supplement: Supplementary file 1 — Supplementary file contains Tables S1-S3 and Figure S1-S8. [file apb-15-521-s001.pdf]

Adv Pharm Bull. 2025;15(3):521-S1
doi: 10.34172/apb.025.45444
https://apb.tbzmed.ac.ir

Supplementary Files

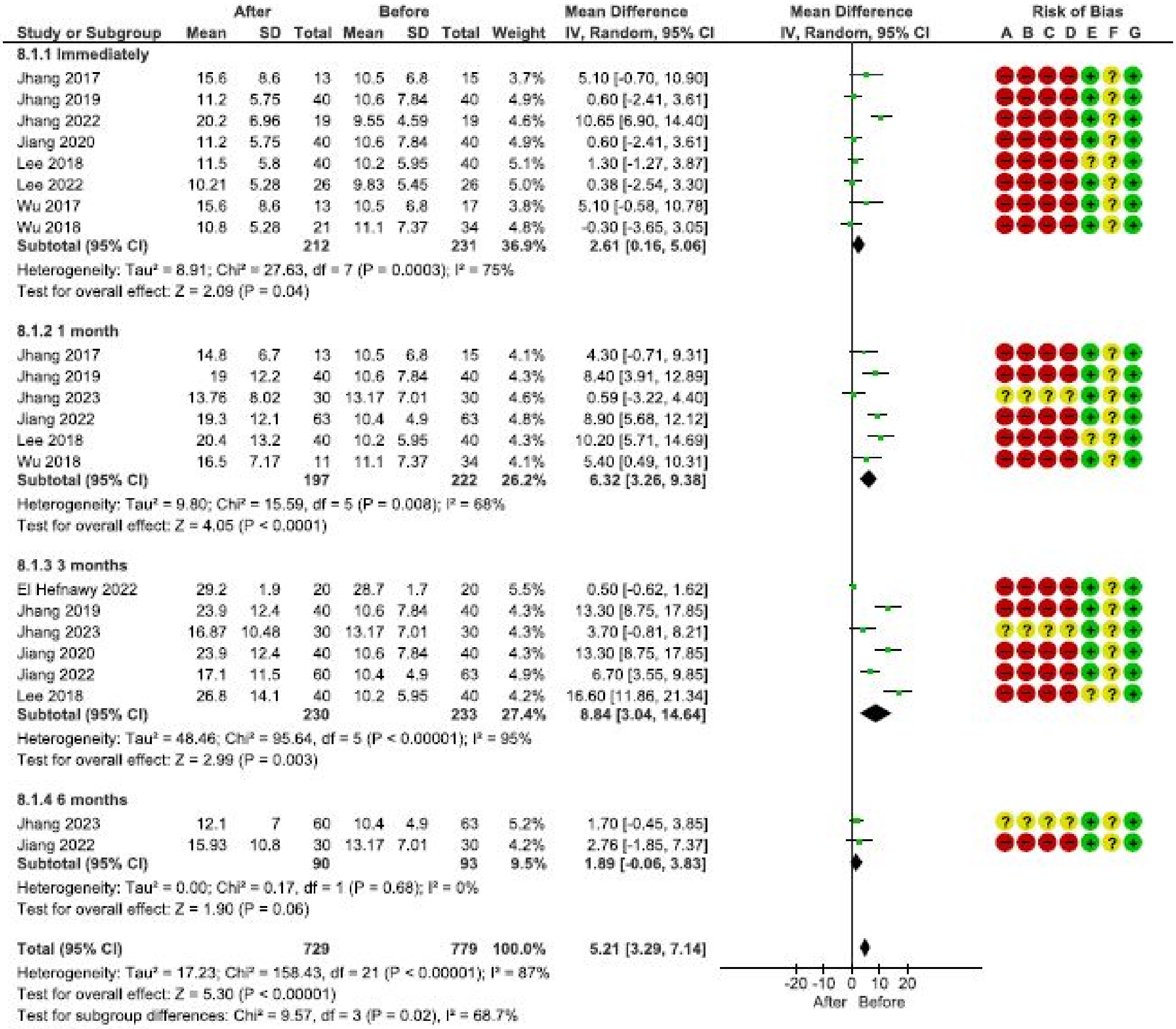

Study or Subgroup	After Mean	After SD	After Total	Before Mean	Before SD	Before Total	Weight	Mean Difference IV, Random, 95% CI	Risk of Bias A B C D E F G
**8.1.1 Immediately**									
Jhang 2017	15.6	8.6	13	10.5	6.8	15	3.7%	5.10 [-0.70, 10.90]	- - - - + ? +
Jhang 2019	11.2	5.75	40	10.6	7.84	40	4.9%	0.60 [-2.41, 3.61]	- - - - + ? +
Jhang 2022	20.2	6.96	19	9.55	4.59	19	4.6%	10.65 [6.90, 14.40]	- - - - + ? +
Jiang 2020	11.2	5.75	40	10.6	7.84	40	4.9%	0.60 [-2.41, 3.61]	- - - - + ? +
Lee 2018	11.5	5.8	40	10.2	5.95	40	5.1%	1.30 [-1.27, 3.87]	- - - - ? ? +
Lee 2022	10.21	5.28	26	9.83	5.45	26	5.0%	0.38 [-2.54, 3.30]	- - - - + ? +
Wu 2017	15.6	8.6	13	10.5	6.8	17	3.8%	5.10 [-0.58, 10.78]	- - - - + ? +
Wu 2018	10.8	5.28	21	11.1	7.37	34	4.8%	-0.30 [-3.65, 3.05]	- - - - + ? +
**Subtotal (95% CI)**			**212**			**231**	**36.9%**	**2.61 [0.16, 5.06]**	
Heterogeneity: Tau² = 8.91; Chi² = 27.63, df = 7 (P = 0.0003); I² = 75%									
Test for overall effect: Z = 2.09 (P = 0.04)									
**8.1.2 1 month**									
Jhang 2017	14.8	6.7	13	10.5	6.8	15	4.1%	4.30 [-0.71, 9.31]	- - - - + ? +
Jhang 2019	19	12.2	40	10.6	7.84	40	4.3%	8.40 [3.91, 12.89]	- - - - + ? +
Jhang 2023	13.76	8.02	30	13.17	7.01	30	4.6%	0.59 [-3.22, 4.40]	? ? ? ? + ? +
Jiang 2022	19.3	12.1	63	10.4	4.9	63	4.8%	8.90 [5.68, 12.12]	- - - - + ? +
Lee 2018	20.4	13.2	40	10.2	5.95	40	4.3%	10.20 [5.71, 14.69]	- - - - ? ? +
Wu 2018	16.5	7.17	11	11.1	7.37	34	4.1%	5.40 [0.49, 10.31]	- - - - + ? +
**Subtotal (95% CI)**			**197**			**222**	**26.2%**	**6.32 [3.26, 9.38]**	
Heterogeneity: Tau² = 9.80; Chi² = 15.59, df = 5 (P = 0.008); I² = 68%									
Test for overall effect: Z = 4.05 (P < 0.0001)									
**8.1.3 3 months**									
El Hefnawy 2022	29.2	1.9	20	28.7	1.7	20	5.5%	0.50 [-0.62, 1.62]	- - - - + ? +
Jhang 2019	23.9	12.4	40	10.6	7.84	40	4.3%	13.30 [8.75, 17.85]	- - - - + ? +
Jhang 2023	16.87	10.48	30	13.17	7.01	30	4.3%	3.70 [-0.81, 8.21]	? ? ? ? + ? +
Jiang 2020	23.9	12.4	40	10.6	7.84	40	4.3%	13.30 [8.75, 17.85]	- - - - + ? +
Jiang 2022	17.1	11.5	60	10.4	4.9	63	4.9%	6.70 [3.55, 9.85]	- - - - + ? +
Lee 2018	26.8	14.1	40	10.2	5.95	40	4.2%	16.60 [11.86, 21.34]	- - - - ? ? +
**Subtotal (95% CI)**			**230**			**233**	**27.4%**	**8.84 [3.04, 14.64]**	
Heterogeneity: Tau² = 48.46; Chi² = 95.64, df = 5 (P < 0.00001); I² = 95%									
Test for overall effect: Z = 2.99 (P = 0.003)									
**8.1.4 6 months**									
Jhang 2023	12.1	7	60	10.4	4.9	63	5.2%	1.70 [-0.45, 3.85]	? ? ? ? + ? +
Jiang 2022	15.93	10.8	30	13.17	7.01	30	4.2%	2.76 [-1.85, 7.37]	- - - - + ? +
**Subtotal (95% CI)**			**90**			**93**	**9.5%**	**1.89 [-0.06, 3.83]**	
Heterogeneity: Tau² = 0.00; Chi² = 0.17, df = 1 (P = 0.68); I² = 0%									
Test for overall effect: Z = 1.90 (P = 0.06)									
**Total (95% CI)**			**729**			**779**	**100.0%**	**5.21 [3.29, 7.14]**	
Heterogeneity: Tau² = 17.23; Chi² = 158.43, df = 21 (P < 0.00001); I² = 87%									
Test for overall effect: Z = 5.30 (P < 0.00001)									
Test for subgroup differences: Chi² = 9.57, df = 3 (P = 0.02), I² = 68.7%									

Risk of bias legend
(A) Random sequence generation (selection bias)
(B) Allocation concealment (selection bias)
(C) Blinding of participants and personnel (performance bias)
(D) Blinding of outcome assessment (detection bias)
(E) Incomplete outcome data (attrition bias)
(F) Selective reporting (reporting bias)
(G) Other bias

Figure S1. The effect of PRP on Maximum flow rate (Qmax) at different time points

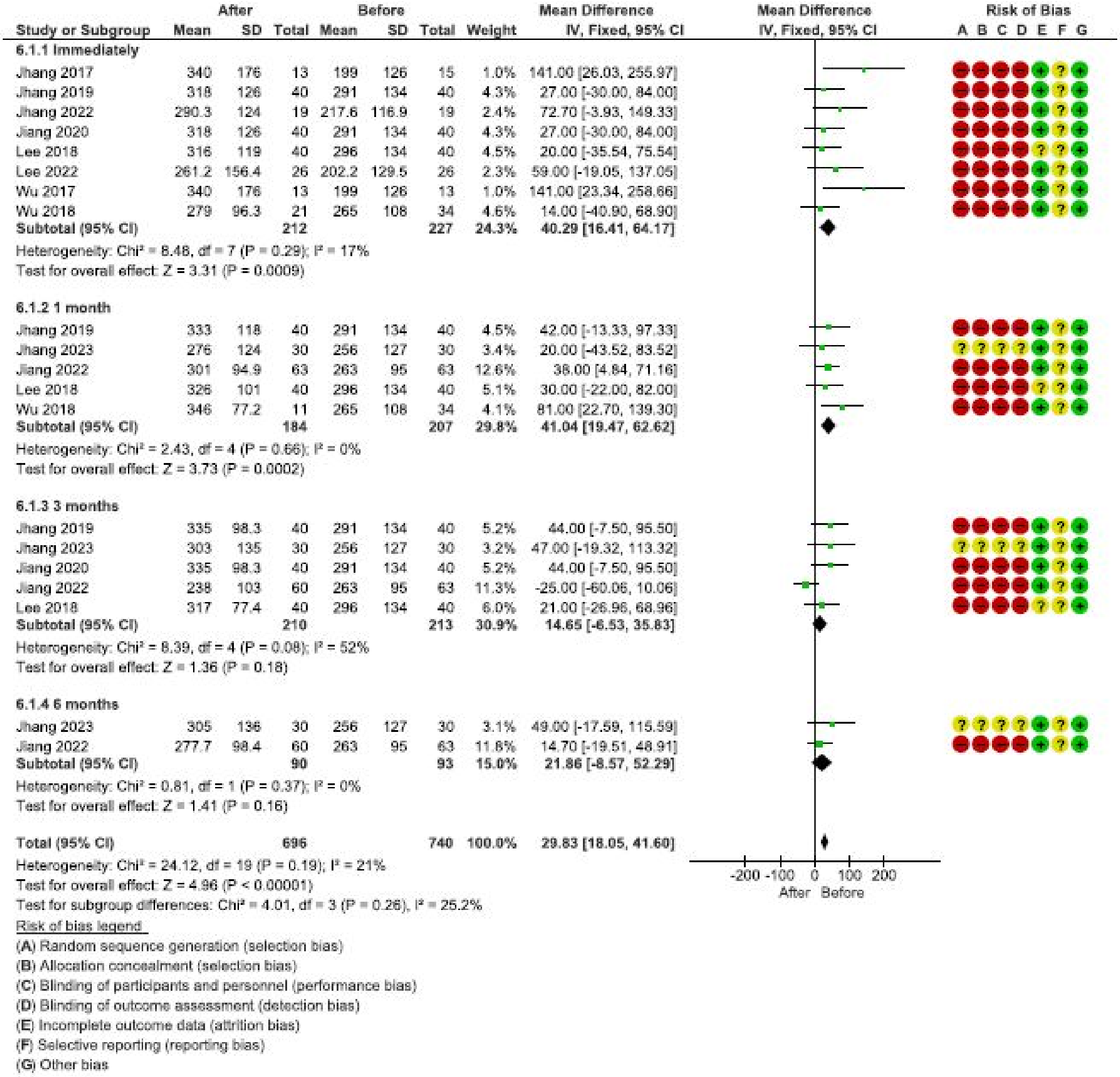

Figure S2. The effect of PRP on Functional bladder capacity at different time points

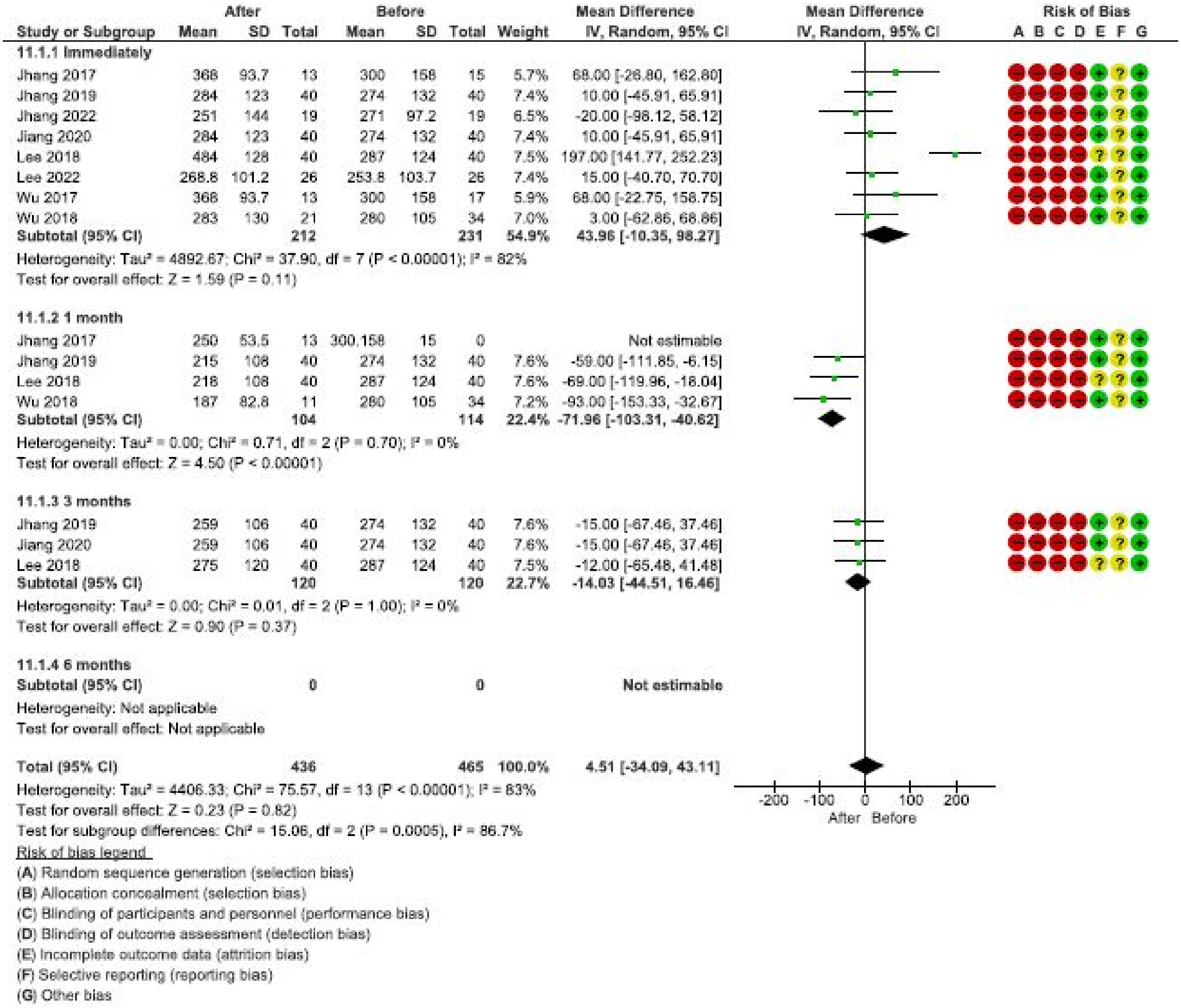

Study or Subgroup	After Mean	After SD	After Total	Before Mean	Before SD	Before Total	Weight	Mean Difference IV, Random, 95% CI
**11.1.1 Immediately**								
Jhang 2017	368	93.7	13	300	158	15	5.7%	68.00 [-26.80, 162.80]
Jhang 2019	284	123	40	274	132	40	7.4%	10.00 [-45.91, 65.91]
Jhang 2022	251	144	19	271	97.2	19	6.5%	-20.00 [-98.12, 58.12]
Jiang 2020	284	123	40	274	132	40	7.4%	10.00 [-45.91, 65.91]
Lee 2018	484	128	40	287	124	40	7.5%	197.00 [141.77, 252.23]
Lee 2022	268.8	101.2	26	253.8	103.7	26	7.4%	15.00 [-40.70, 70.70]
Wu 2017	368	93.7	13	300	158	17	5.9%	68.00 [-22.75, 158.75]
Wu 2018	283	130	21	280	105	34	7.0%	3.00 [-62.86, 68.86]
**Subtotal (95% CI)**			**212**			**231**	**54.9%**	**43.96 [-10.35, 98.27]**
Heterogeneity: Tau² = 4892.67; Chi² = 37.90, df = 7 (P < 0.00001); I² = 82%								
Test for overall effect: Z = 1.59 (P = 0.11)								
**11.1.2 1 month**								
Jhang 2017	250	53.5	13	300,158	15	0		Not estimable
Jhang 2019	215	108	40	274	132	40	7.6%	-59.00 [-111.85, -6.15]
Lee 2018	218	108	40	287	124	40	7.6%	-69.00 [-119.96, -18.04]
Wu 2018	187	82.8	11	280	105	34	7.2%	-93.00 [-153.33, -32.67]
**Subtotal (95% CI)**			**104**			**114**	**22.4%**	**-71.96 [-103.31, -40.62]**
Heterogeneity: Tau² = 0.00; Chi² = 0.71, df = 2 (P = 0.70); I² = 0%								
Test for overall effect: Z = 4.50 (P < 0.00001)								
**11.1.3 3 months**								
Jhang 2019	259	106	40	274	132	40	7.6%	-15.00 [-67.46, 37.46]
Jiang 2020	259	106	40	274	132	40	7.6%	-15.00 [-67.46, 37.46]
Lee 2018	275	120	40	287	124	40	7.5%	-12.00 [-65.48, 41.48]
**Subtotal (95% CI)**			**120**			**120**	**22.7%**	**-14.03 [-44.51, 16.46]**
Heterogeneity: Tau² = 0.00; Chi² = 0.01, df = 2 (P = 1.00); I² = 0%								
Test for overall effect: Z = 0.90 (P = 0.37)								
**11.1.4 6 months**								
**Subtotal (95% CI)**			**0**			**0**		**Not estimable**
Heterogeneity: Not applicable								
Test for overall effect: Not applicable								
**Total (95% CI)**			**436**			**465**	**100.0%**	**4.51 [-34.09, 43.11]**

Heterogeneity: Tau² = 4406.33; Chi² = 75.57, df = 13 (P < 0.00001); I² = 83%
Test for overall effect: Z = 0.23 (P = 0.82)
Test for subgroup differences: Chi² = 15.06, df = 2 (P = 0.0005), I² = 86.7%

Risk of bias legend
(A) Random sequence generation (selection bias)
(B) Allocation concealment (selection bias)
(C) Blinding of participants and personnel (performance bias)
(D) Blinding of outcome assessment (detection bias)
(E) Incomplete outcome data (attrition bias)
(F) Selective reporting (reporting bias)
(G) Other bias

Figure S3. The effect of PRP on Cystometric bladder capacity at different time points

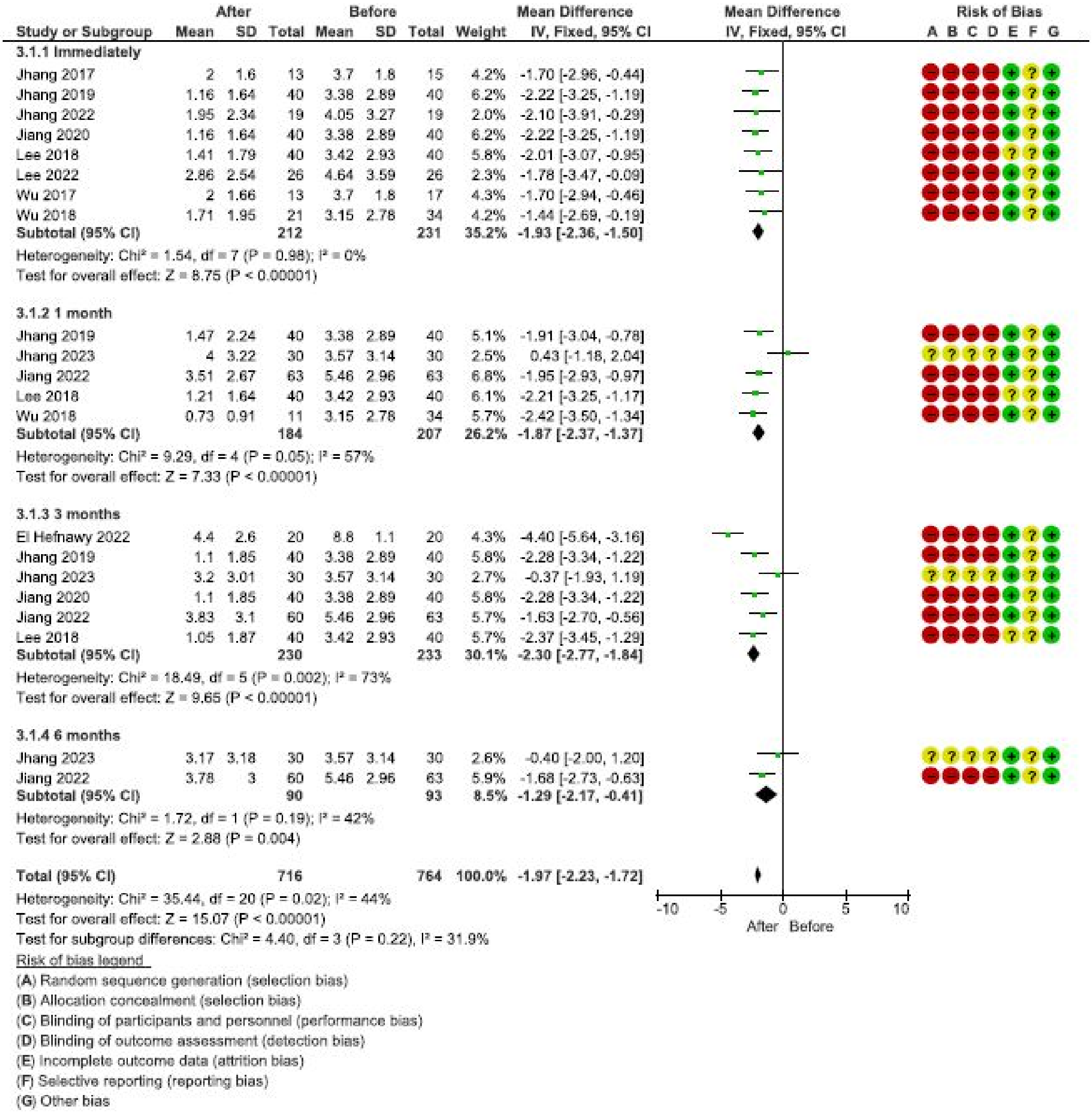

Figure S4. The effect of PRP on VAS score at different time points

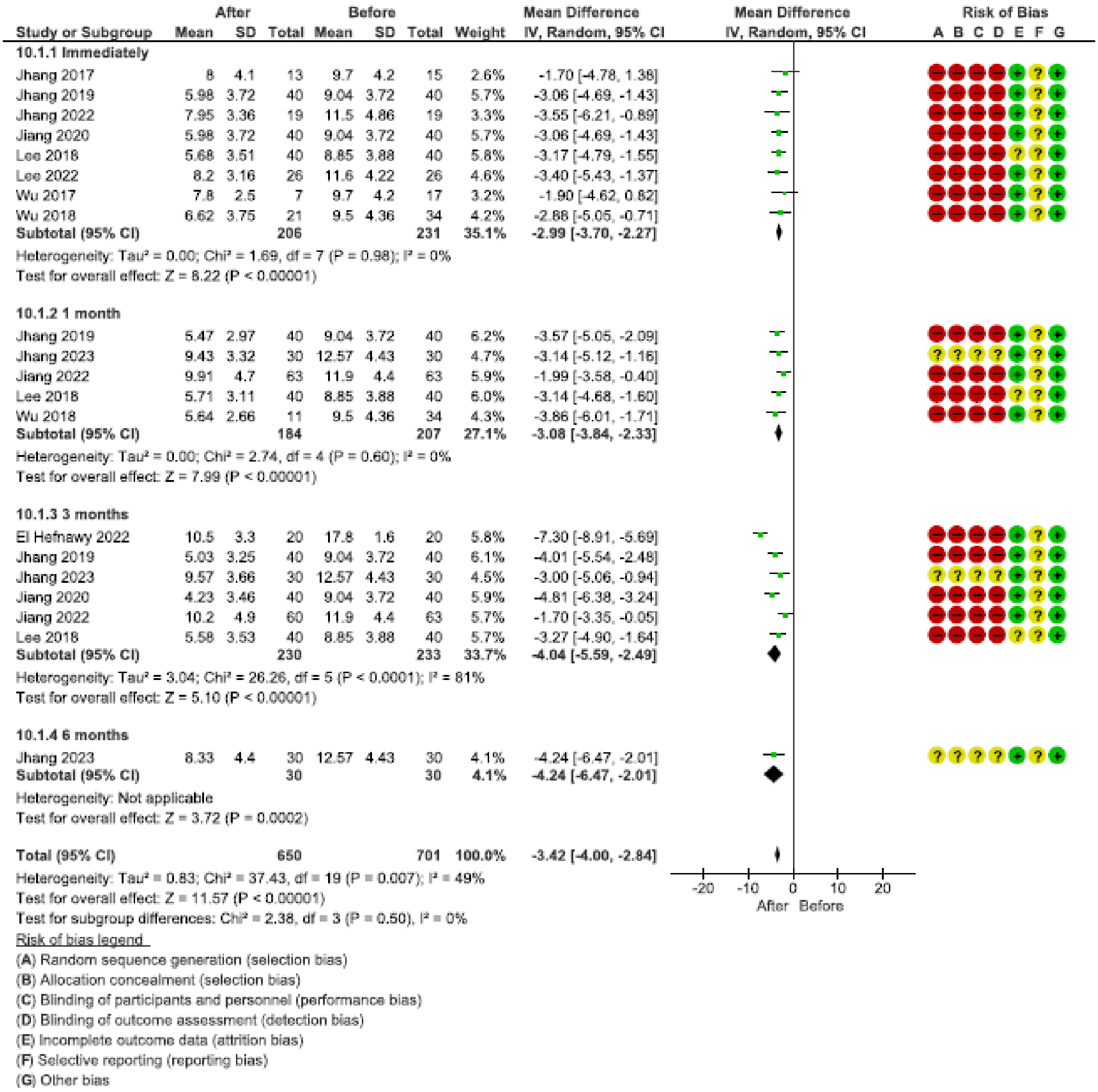

Figure S5. The effect of PRP on ICSI score at different time points

Study or Subgroup	After Mean	After SD	After Total	Before Mean	Before SD	Before Total	Weight	Mean Difference IV, Random, 95% CI
**12.1.1 Immediately**								
Jhang 2017	6.5	4.6	13	9.7	3.6	15	3.3%	-3.20 [-6.29, -0.11]
Jhang 2019	6.44	4.07	40	9.8	3.6	40	5.3%	-3.36 [-5.04, -1.68]
Jhang 2022	6.58	3.5	19	9.47	4.17	19	4.1%	-2.89 [-5.34, -0.44]
Jiang 2020	6.44	4.07	40	9.8	3.6	40	5.3%	-3.36 [-5.04, -1.68]
Lee 2018	6.88	4.18	40	9.73	3.73	40	5.2%	-2.85 [-4.59, -1.11]
Lee 2022	8.7	3.77	26	10.7	3.37	26	4.9%	-2.00 [-3.94, -0.06]
Wu 2017	8.4	3.5	7	9.7	3.6	17	3.3%	-1.30 [-4.41, 1.81]
Wu 2018	7.71	4.2	21	10.2	3.87	34	4.5%	-2.49 [-4.71, -0.27]
**Subtotal (95% CI)**			**206**			**231**	**35.9%**	**-2.82 [-3.55, -2.09]**
Heterogeneity: Tau² = 0.00; Chi² = 2.54, df = 7 (P = 0.92); I² = 0%								
Test for overall effect: Z = 7.58 (P < 0.00001)								
**12.1.2 1 month**								
Jhang 2019	6	3.3	40	9.8	3.6	40	5.5%	-3.80 [-5.31, -2.29]
Jhang 2023	8.93	3.54	30	11.63	3.21	30	5.2%	-2.70 [-4.41, -0.99]
Jiang 2022	10.2	4.4	63	12.3	3.4	63	5.7%	-2.10 [-3.47, -0.73]
Lee 2018	6.58	3.35	40	9.73	3.73	40	5.5%	-3.15 [-4.70, -1.60]
Wu 2018	6.36	3.75	11	10.2	3.87	34	4.0%	-3.84 [-6.41, -1.27]
**Subtotal (95% CI)**			**184**			**207**	**26.0%**	**-2.98 [-3.71, -2.25]**
Heterogeneity: Tau² = 0.00; Chi² = 3.28, df = 4 (P = 0.51); I² = 0%								
Test for overall effect: Z = 7.99 (P < 0.00001)								
**12.1.3 3 months**								
El Hefnawy 2022	8	2.8	20	13	1.4	20	5.7%	-5.00 [-6.37, -3.63]
Jhang 2019	4.23	3.46	40	9.8	3.6	40	5.5%	-5.57 [-7.12, -4.02]
Jhang 2023	8.87	3.21	30	11.63	3.21	30	5.4%	-2.76 [-4.38, -1.14]
Jiang 2020	5.03	3.25	40	9.8	3.6	40	5.5%	-4.77 [-6.27, -3.27]
Jiang 2022	12.3	3.4	63	10.6	4.7	60	5.6%	1.70 [0.24, 3.16]
Lee 2018	5	3.5	40	9.73	3.79	40	5.4%	-4.73 [-6.33, -3.13]
**Subtotal (95% CI)**			**233**			**230**	**33.1%**	**-3.52 [-5.77, -1.27]**
Heterogeneity: Tau² = 7.29; Chi² = 66.29, df = 5 (P < 0.00001); I² = 92%								
Test for overall effect: Z = 3.07 (P = 0.002)								
**12.1.4 6 months**								
Jhang 2023	8.6	4.06	30	11.63	3.21	30	5.0%	-3.03 [-4.88, -1.18]
**Subtotal (95% CI)**			**30**			**30**	**5.0%**	**-3.03 [-4.88, -1.18]**
Heterogeneity: Not applicable								
Test for overall effect: Z = 3.21 (P = 0.001)								
**Total (95% CI)**			**653**			**698**	**100.0%**	**-3.09 [-3.88, -2.31]**
Heterogeneity: Tau² = 2.29; Chi² = 74.29, df = 19 (P < 0.00001); I² = 74%								
Test for overall effect: Z = 7.75 (P < 0.00001)								
Test for subgroup differences: Chi² = 0.38, df = 3 (P = 0.94), I² = 0%								

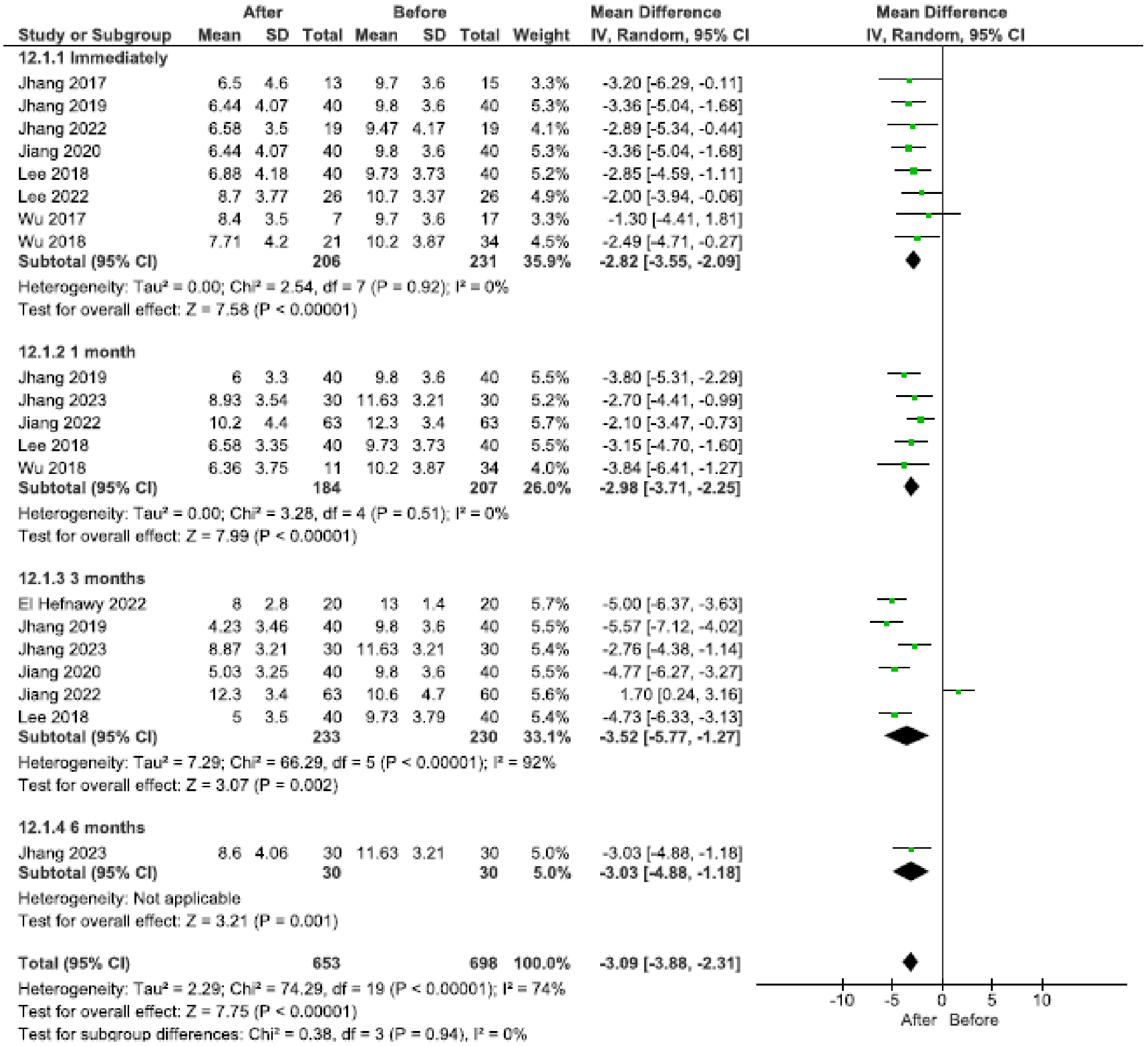

Figure S6. The effect of PRP on ICPI score at different time points

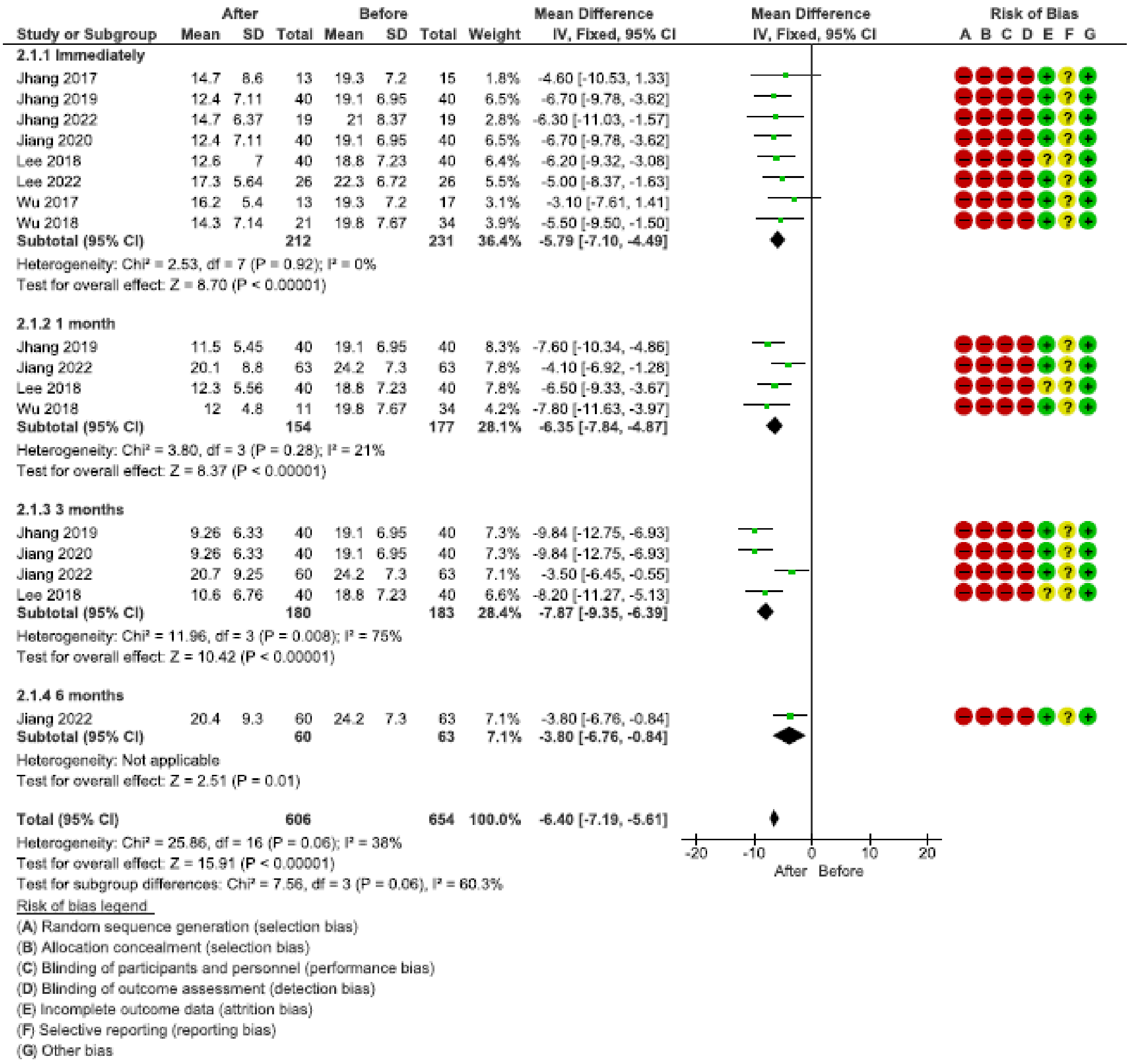

Figure S7. The effect of PRP on OSS at different time points

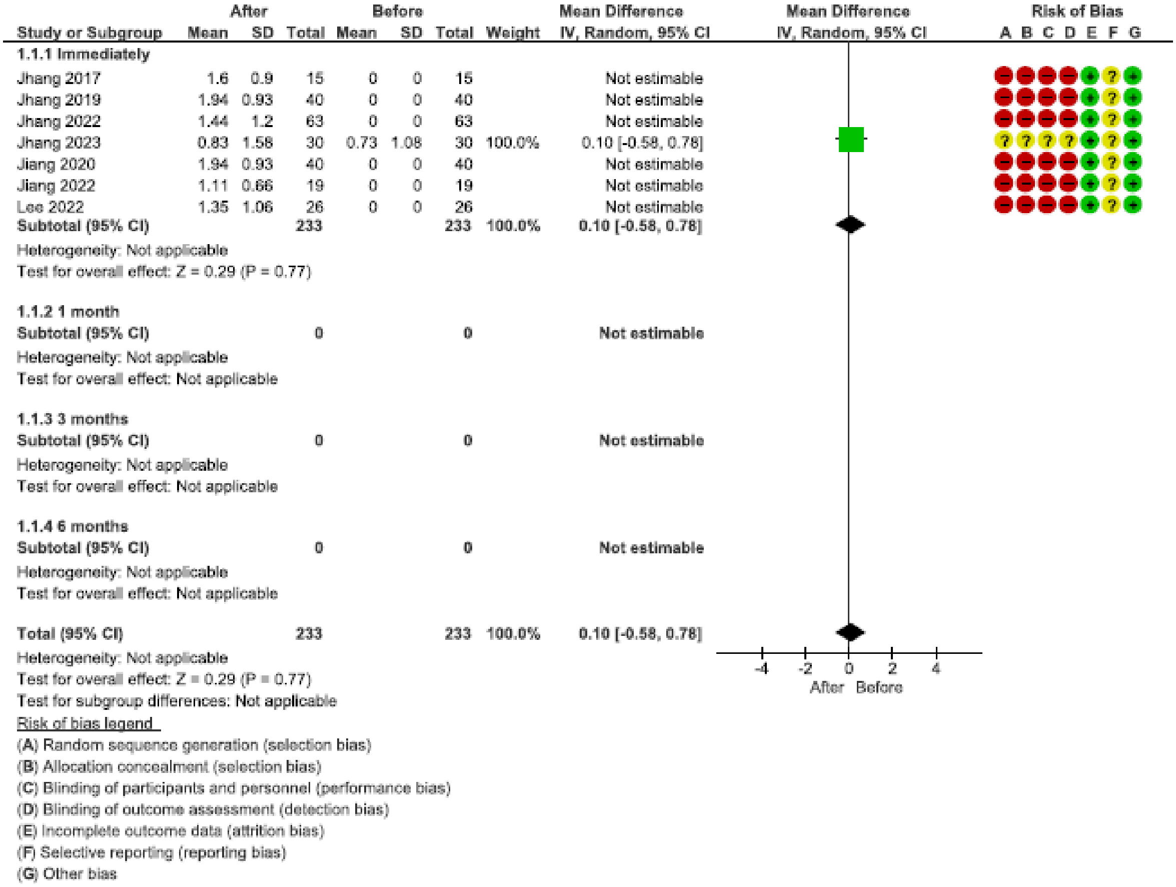

Figure S8. The effect of PRP on GRA at different time points

Supplementary File 1. Search strategy in PubMed				
Search number	Query	Sort By	Filters	Results
3	((((((((("Platelet-Rich Plasma"[Mesh]) OR ((Platelet[Text Word] AND Rich[Text Word] AND Plasma[Text Word]))) OR (Platelet-rich plasma[Text Word])) OR ((platelet[Text Word] AND enrich*[Text Word] AND plasma[Text Word]))) OR (Platelet-Rich Fibrin[Text Word])) OR ((Platelet[Text Word] AND Rich[Text Word] AND Fibrin[Text Word]))) OR (PRP[Text Word])) OR (Autologous conditioned plasma[Text Word])) AND ((((((((((("Cystitis, Interstitial"[Mesh]) OR ("Prostatitis"[Mesh])) OR ("Pelvic Pain"[Mesh])) OR ((Interstitial[Text Word] AND Cystitis[Text Word]))) OR ((Interstitial[Text Word] AND Cystitides[Text Word]))) OR ((Bladder[Text Word] AND Pain*[Text Word] AND Syndrome*[Text Word]))) OR ("Interstitial Cystitis/Bladder Pain Syndrome"[Text Word])) OR ("IC/BPS"[Text Word])) OR ((pelvi*[Text Word] AND pain*[Text Word]))) OR (Prostatitides[Text Word])) OR (Prostatitis[Text Word]))			44
2	((((((((((("Cystitis, Interstitial"[Mesh]) OR ("Prostatitis"[Mesh])) OR ("Pelvic Pain"[Mesh])) OR ((Interstitial[Text Word] AND Cystitis[Text Word]))) OR ((Interstitial[Text Word] AND Cystitides[Text Word]))) OR ((Bladder[Text Word] AND Pain*[Text Word] AND Syndrome*[Text Word]))) OR ("Interstitial Cystitis/Bladder Pain Syndrome"[Text Word])) OR ("IC/BPS"[Text Word])) OR ((pelvi*[Text Word] AND pain*[Text Word]))) OR (Prostatitides[Text Word])) OR (Prostatitis[Text Word])			49,086
1	((((((("Platelet-Rich Plasma"[Mesh]) OR ((Platelet[Text Word] AND Rich[Text Word] AND Plasma[Text Word]))) OR (Platelet-rich plasma[Text Word])) OR ((platelet[Text Word] AND enrich*[Text Word] AND plasma[Text Word]))) OR (Platelet-Rich Fibrin[Text Word])) OR ((Platelet[Text Word] AND Rich[Text Word] AND Fibrin[Text Word]))) OR (PRP[Text Word])) OR (Autologous conditioned plasma[Text Word])			29,872

Supplementary File 2

Table S1. **Characteristics of the included studies**

Citation				Intervention							Intervention group									
	**Country**	**Design**	**Area of bladder treated**	**Type**	**Number of injections**	**Dose**	**Frequency**	**Route of administration**	**Blood sample**	**Duration**	**Number**	**Condition**	**Age**	**Gender (F/M)**	**Duration of disease**	**Control**	**F/U**	**A/E**	**Main outcomes**	**Overall results**
**Jhang (2019)**[22]	Taiwan	Prospective clinical trial	Posterior and lateral walls of the bladder	PRP	20	10 mL	Monthly	Intravesical, suburothelium	50 mL whole blood	4 months	40	IC/BPS who had failed conventional treatment	55.5 ± 11.1	37/3	In GRA ≥ 2 group: 12.6 ± 9.01 years; In GRA < 2 group: 17.2 ± 8.59 years	None	Every month nd 3 months after the 4th PRP treatment day. They were also followed up for the therapeutic duration for up to 12	None	Global Response Assessment (GRA), O'Leary-Sant symptom score (OSS), visual analog scale (VAS) of pain, daily frequency, nocturia, functional bladder capacity (FBC), maximum flow rate, voided volume, post-void residual volume (PVR)	The study demonstrated that repeated intravesical injections of autologous PRP can increase bladder capacity and provide IC symptom improvement in patients with IC/BPS refractory to conventional therapy. Autologous PRP injection is safe and effective in selected patients.

**Lee (2022)**[27]	Taiwan	Prospective clinical trial	Posterior and lateral walls of the bladder	PRP	4	10 mL	Monthly	Intravesical, suburothelium	50 mL whole blood	4 months	26	Refractory non-ulcer IC/BPS	58.6 ± 14.2	NR	NR	None	4 months	NR	functional bladder capacity (FBC), urinary frequency and number of nocturia episode، A 10-point Visual Analog Scale (VAS) to evaluate the severity of bladder pain and the O'Leary–Sant symptom score (OSS) [illegible]	Repeated intravesical PRP injections are effective for improving IC/BPS symptoms as they promote urothelial ultrastructural defect recovery.
**Jiang (2022)**[24]	Taiwan	Prospective clinical trial	Posterior and lateral walls of the bladder	High and low dose PRP with different preparation and injection sites	1	10 mL	Once	intravesical	100 ml of whole blood	Once	60	IC/BPS	NR	NR	NR	None	1 month, 3 months (3 months after single high-dose PRP and before 4th low-dose PRP), and 6 months (6 months after single high-dose PRP and 3 months after 4th low-dose PRP)	No AEs related to PRP injections, such as UTIs or acute urinary retention, occurred throughout the current study	IC symptom index (ICSI) and problem index (ICPI), visual analog scale (VAS), global response assessment (GRA), and urodynamic parameters.	Intravesical PRP injection is effective for IC/BPS. The addition of normal saline or plasma and injection site had no influence on therapeutic efficacy. However, the symptom improvement and GRA after a single high-dose PRP injection was lower than that after four low-dose PRP [illegible]
**Jhang (2019)**[31]	Taiwan	Prospective clinical trial	Posterior and lateral walls of the bladder	PRP	4	12 mL	Monthly	Suburothelial	50 mL whole blood	4 months	13	IC/BPS was refractory to conventional treatments	52.9 ± 12.1	All F	NR	None	1 month	NR	Primary: change in O'Leary-Sant symptom (OSS) index; Secondary: pain (measured using a visual analog scale [VAS]), daily frequency, nocturia, functional bladder capacity (FBC), maximum flow rate, voided volume, post-void residual (PVR) volume, and global response assessment (GRA). ICSI, ICPI, cytokine level	Repeated intravesical PRP injections are well tolerated and appear to be safe and effective in medically refractive IC/BPS, providing significant symptom improvement

**Jhang (2023)[23]**	Taiwan	Prospective cohort	20 well-distributed sites at posterior and lateral bladder wall	PRP	4	NR	Monthly	?	?	4 months	30	IC/BPS who were refractory to conventional treatment	52.57 ± 11.08	All F	10.27 ± 8.85 years	IC/BPS who were refractory to conventional treatment and received BoNT-A (n = 26)	6 month	patients in the BoNT-A group complained of dysuria after treatment, and a significantly higher rate of urinary tract	Primary: global response assessment (GRA), secondary outcome: changes in the O'Leary-Sant IC symptom score, visual analog score (VAS) of bladder pain, voiding diary, and uroflow measures	Both intravesical PRP and BoNT-A injections have similar efficacy in IC symptom improvement.
**Jiang (2020)[25]**	Taiwan	Prospective clinical trial	Posterior and lateral walls	PRP	4	10 mL	Monthly	Intravesical	peripheral blood platelet concentration	4 months	40	IC/BPS	55.5 ± 11.1	37/3	NR		3 months after the last injection		The clinical parameters included visual analog scale (VAS) pain score, daily urinary frequency, nocturia episodes, functional bladder capacity, and global response assessment (GRA).	Repeated intravesical PRP injections provided significant symptom improvement in IC/BPS patients with concomitant changes in the related biomarker levels
**El Hefnawy (2022)[19]**	Egypt	Prospective clinical trial	?	PRP	6	50 mL	Weekly	Intravesical	150 mL of blood sample	12 weeks	20	IC/BPS	38.7 ± 10	All F	NR	None	12 weeks	All the patients reported discomfort with blood sample withdrawal, and no other adverse events were	The primary: visual analog scale (VAS) for pain; the secondary endpoints: the IC symptom index, IC problem index of the O'Leary-Sant questionnaire and global response assessment, urine culture, and uroflowmetry.	Repeated intravesical instillation of PRP could be considered an effective and safe approach for treating IC/BPS
**Jhang (2022)[21]**	Taiwan	?	posterior and lateral walls of the	PRP	4	NR	Monthly	Intravesical	? mL of peripheral blood	4 months	19	IC/BPS without Hunner's	55.6 ± 15.8	16/3	NR	None	7–10 days after fourth PRP injection	NR	Changes in the O'Leary-Sant symptom score (OSS) including IC symptom index (ICSI) and IC	The level of urothelial barrier function protein and cell [illegible]

**Hung (2022)**[20]	Taiwan	Prospective clinical trial	NR	Nanofat grafts + PRP		8 mL	Once	?	20-mL whole blood	Once	6	Refractory IC/BPS	46.3 ± 4.7	All F	24.7 ± 13.6 months	None	6 months	No significant AE	GRA, ICSI, ICPI, Pain-VAS, Cystoscopic capacity	Our preliminary results suggest novel intravesical therapy with autologous Nanofat plus PRP grafting is safe and effective for refractory IC/BPS
**Studies without access to full text**																				
**Wu (2017)**[29]	Taiwan	Prospective clinical trial	?	PRP	4	10-12 mL	Monthly	intravesical	50ml of patient's own whole blood	?	13	Refractory IC/BPS	52.9 ± 12.1	All F	?	None	1 month after the 4th injection	All patients were free of urinary tract infection or [illegible]	Primary end-point was the change of the O'Leary-Sant symptom score (OSS, including ICSI and ICPI) from [illegible]	The results of this study demonstrates that intravesical injections of [illegible]
**Wu (2018)**[30]	Taiwan	Prospective clinical trial	?	PRP	4	12 mL	Monthly	Intravesical	50 mL of the patient's whole blood	?	34	IC/BPS	53.8 ± 12.3	31/3	?	None	1 month after the 4th injection	?	O'Leary-Sant symptom (OSS), the IC symptom index (ICSI), and IC problem index (ICPI) , pain score [illegible]	Intravesical PRP injection is a possible effective treatment for medically [illegible]
**Lee (2018)**[26]	Taiwan	Prospective clinical trial	?	PRP	4	12 mL	Monthly	Intravesical	50 mL of the patient's whole blood		40	IC/BPS	55.5 ± 11.1	37/3	?	None	1 and 3 months after the last injection	All the patients were free of infection and [illegible]	GRA, OSS, VAS, bladder function and urodynamic measure	Repeated PRP injection is a safe, well-tolerated and sfae therapeutic [illegible]
**Medvedev (2023)**[28]		Prospective RCT	?	PRP	5	?	Once in 2 weeks	intradetrusor	?	10 weeks	85	IC/BPS	20 to 79	All F	2.7 – 6.9	35 IC/BPS women received a [illegible]	?	?	pain (VAS-scale), urgency and frequency (PUF-scale), bladder diary and bladder capacity (bladder [illegible]	Standard IC / BPS therapy leads to a significant decrease in the [illegible]

Table S2.SOF Table and GRADE Recommendations

Outcome	Importance	No. of Studies (Participants)	Quality of Evidence (GRADE)	Findings (MD [95% CI])	Interpretation
**Urinary Frequency**	Critical	7 (677)	**Low (⊕⊕⊖⊖) ⬇**	-1.97 [-2.52, -1.42]	Likely reduces frequency, but uncontrolled studies weaken certainty.
**Nocturia**	Critical	6 (606)	**Low (⊕⊕⊖⊖) ⬇**	-0.51 [-0.66, -0.36]	Likely reduces nocturia, but uncontrolled studies inflate effect.
**Voided Volume**	Important	5 (729)	**Very Low (⊕⊖⊖⊖) ⬇⬇**	15.36 [4.03, 26.69]	Unclear due to high heterogeneity and lack of controls.
**PVR**	Important	8 (779)	**Very Low (⊕⊖⊖⊖) ⬇⬇**	-16.87 [-26.51, -7.24]	Unclear due to inconsistency and bias.
**Qmax**	Important	8 (779)	**Very Low (⊕⊖⊖⊖) ⬇⬇**	5.21 [3.29, 7.14]	Unclear due to high heterogeneity and no control comparison.
**FBC**	Important	6 (716)	**Low (⊕⊕⊖⊖) ⬇**	29.83 [18.05, 41.60]	Likely improves FBC, but uncontrolled studies limit certainty.
**CBC**	Important	5 (725)	**Very Low (⊕⊖⊖⊖) ⬇⬇**	0.18 [-15.61, 15.97]	No clear effect; very low confidence.
**VAS (Pain)**	Critical	8 (716)	**Low (⊕⊕⊖⊖) ⬇**	-1.93 [-2.28, -1.58]	Likely reduces pain, but placebo effect cannot be ruled out.
**OSS**	Critical	6 (606)	**Low (⊕⊕⊖⊖) ⬇**	-6.35 [-7.36, -5.33]	Likely improves symptoms, but bias is a concern.

**ICSI**	Important	5 (606)	**Low (⊕⊕⊖⊖) ⬇**	-4.24 [-6.47, -2.01]	Likely reduces symptoms, but uncontrolled studies weaken certainty.
**ICPI**	Important	5 (606)	**Low (⊕⊕⊖⊖) ⬇**	-3.09 [-3.88, -2.31]	Likely reduces pain, but placebo effect possible.
